# Prior fear conditioning and reward learning interact in fear and reward networks

**DOI:** 10.3389/fnbeh.2014.00067

**Published:** 2014-03-04

**Authors:** Lisa Bulganin, Dominik R. Bach, Bianca C. Wittmann

**Affiliations:** ^1^Department of Psychology and Sports Science, University of GiessenGiessen, Germany; ^2^Psychiatric Hospital, University of ZurichZurich, Switzerland; ^3^Wellcome Trust Centre for Neuroimaging, University College LondonLondon, UK

**Keywords:** fear, reward, counterconditioning, striatum, fMRI, skin conductance response

## Abstract

The ability to flexibly adapt responses to changes in the environment is important for survival. Previous research in humans separately examined the mechanisms underlying acquisition and extinction of aversive and appetitive conditioned responses. It is yet unclear how aversive and appetitive learning interact on a neural level during counterconditioning in humans. This functional magnetic resonance imaging (fMRI) study investigated the interaction of fear conditioning and subsequent reward learning. In the first phase (fear acquisition), images predicted aversive electric shocks or no aversive outcome. In the second phase (counterconditioning), half of the CS+ and CS− were associated with monetary reward in the absence of electric stimulation. The third phase initiated reinstatement of fear through presentation of electric shocks, followed by CS presentation in the absence of shock or reward. Results indicate that participants were impaired at learning the reward contingencies for stimuli previously associated with shock. In the counterconditioning phase, prior fear association interacted with reward representation in the amygdala, where activation was decreased for rewarded compared to unrewarded CS− trials, while there was no reward-related difference in CS+ trials. In the reinstatement phase, an interaction of previous fear association and previous reward status was observed in a reward network consisting of substantia nigra/ventral tegmental area (SN/VTA), striatum and orbitofrontal cortex (OFC), where activation was increased by previous reward association only for CS− but not for CS+ trials. These findings suggest that during counterconditioning, prior fear conditioning interferes with reward learning, subsequently leading to lower activation of the reward network.

## Introduction

The ability to learn about threats and rewards in the environment and flexibly adapt behavior to changed action outcomes is important for survival. A large number of studies have addressed the underlying processes in learning about aversive outcomes using classical fear conditioning procedures. In fear conditioning paradigms, a neutral stimulus (the conditioned stimulus, CS+) is repeatedly paired with an aversive outcome (the unconditioned stimulus, UCS). Learning the association between CS+ and UCS results in a fear response to the CS+ (conditioned response, CR), which is not elicited by an unpaired neutral stimulus (CS−). Human functional magnetic resonance imaging (fMRI) studies showed that the amygdala, anterior cingulate cortex (ACC), and insular cortex are involved in the acquisition of this conditioned response (for a review, see Sehlmeyer et al., 2009). More recently, attention has been brought to the mechanisms underlying adaptive reductions in fear (for a review, see Hartley and Phelps, 2010), such as those induced by extinction procedures in which the CS+ is presented in the absence of the UCS. Extinction learning involves the amygdala, ventromedial prefrontal cortex (vmPFC) and hippocampus (for reviews, see Sehlmeyer et al., 2009; Hartley and Phelps, 2010; Milad and Quirk, 2012; Vervliet et al., 2013).

Extinguished fear responses have been shown to return under various conditions, such as after a context change (renewal), after the passage of time (spontaneous recovery), and after unsignaled presentation of the UCS (reinstatement). In fear reinstatement procedures, fear acquisition and extinction are followed by a third phase, in which unreinforced presentation of CS is typically preceded by several unexpected presentations of the UCS (Rescorla and Heth, 1975; Bouton and Bolles, 1979). Recent reinstatement experiments in humans indicated that reinstatement is context dependent, occurs with various timings after acquisition and extinction, and also occurs after presentation of aversive stimuli different from the original UCS (Vervliet et al., 2013). A recent fMRI study reported an involvement of vmPFC and hippocampus in reinstatement of fear (Lonsdorf et al., 2013).

In contrast to extinction, very little is known about the neural mechanisms underlying fear reduction through counterconditioning. In counterconditioning procedures, a CS is successively paired with qualitatively different UCSs (aversive, appetitive) in consecutive phases of the experiment. Previous studies reported that appetitive conditioning to a learned fear CS resulted in enhanced fear reduction in comparison to other procedures modifying conditioned fear (Dickinson and Pearce, 1977; Raes and De Raedt, 2012). In addition to this influence of reward on the acquired fear response, effects of the learned fear association on reward learning to the fear CS+ have also been investigated. There is evidence that prior fear conditioning can delay the development of an appetitive CR to the fear CS+ in a subsequent appetitive conditioning phase (Scavio, 1974; Bromage and Scavio, 1978; Krank, 1985).

The neural mechanisms of these interactions between appetitive and aversive learning have not been explored yet. The amygdala, vmPFC and hippocampus are candidate regions, since these regions are involved in the development and retention of fear extinction (Sehlmeyer et al., 2009; Hartley and Phelps, 2010; Milad and Quirk, 2012; Vervliet et al., 2013). Reward processing additionally involves the dopaminergic midbrain, striatum and orbitofrontal cortex (OFC) (Levy and Glimcher, 2012). Moreover, recent studies suggest substantial overlap between fear and reward networks. Among others, the amygdala (Belova et al., 2007; Morrison and Salzman, 2010; Fernando et al., 2013), midbrain dopamine neurons (Matsumoto and Hikosaka, 2009) and striatum (Delgado et al., 2008b; Wittmann et al., 2013) have been shown to be responsive to both rewards and different types of punishments including those used in classical fear conditioning. These regions are therefore ideally suited to integrate appetitive and aversive signals during counterconditioning. In line with this hypothesis, a previous study demonstrated that expectation of pain decreased reward sensitivity in striatum and ACC during goal-directed behavior in an explicit decision-making task involving mixed pain and reward outcomes (Talmi et al., 2009).

The studies described above have demonstrated interactions of fear and reward processing during counterconditioning. It is yet unclear to what extent these mechanisms influence later reinstatement of fear. In rats, occurrence of fear reinstatement has been demonstrated after counterconditioning, suggesting parallels between counterconditioning and extinction mechanisms (Brooks et al., 1995). We hypothesized that stronger fear reduction by counterconditioning (Dickinson and Pearce, 1977; Raes and De Raedt, 2012) could potentially be more resistant to subsequent reinstatement.

The aims of the current study were to investigate (i) the effect of counterconditioning on acquired fear responses, (ii) the influence of acquired fear association on reward learning to fear CS+, and (iii) the effects of prior counterconditioning on reinstatement of fear. We addressed these questions in an fMRI design consisting of three phases. In the first phase (fear acquisition), participants underwent a delay fear conditioning procedure in which visual cues were associated with electric shocks. Four stimuli (CS+) were probabilistically coupled to the UCS, while two stimuli (CS−) were never paired with the UCS. In the second phase (counterconditioning), fear extinction was combined with counterconditioning. Half of the previous CS+ and CS− were probabilistically reinforced with monetary reward, while the other half was followed by neutral outcomes. This allowed a comparison of rewarded and unrewarded trials as a function of prior fear association. To investigate return of fear, the third phase initiated fear reinstatement by presenting four unexpected reminder shocks, followed by unreinforced CS presentation (no shock or reward). In accordance with the majority of prior reinstatement studies in humans, all three phases were presented on one testing day to allow comparison to prior results (for a review, see Vervliet et al., 2013). Our fMRI hypotheses were specific to the task phases. To confirm successful fear conditioning, activation in amygdala, ACC and insular cortex in the acquisition phase was analyzed (Sehlmeyer et al., 2009). The main fMRI analysis focused on brain regions relevant to fear extinction (amygdala, vmPFC, hippocampus; Hartley and Phelps, 2010; Schiller and Delgado, 2010) and reward learning (SN/VTA, caudate nucleus, putamen, OFC; Haber and Knutson, 2010). We further hypothesized that a decrease of conditioned skin conductance responses (SCR) during fear extinction would be enhanced by counterconditioning (Raes and De Raedt, 2012), and SCR were expected to increase following the reminder shocks at the beginning of the reinstatement phase (Labar and Phelps, 2005; Sokol and Lovibond, 2012). Throughout this text, we will use the terms CS+ and CS− to refer to fear predictiveness acquired in the first (fear acquisition) phase of the experiment, but not to the reward predictiveness acquired in the counterconditioning phase, which will be indicated by the terms “rewarded”/”unrewarded.”

## Experimental procedures

### Participants

Thirty-eight healthy, right-handed adults participated in the study. Data of 12 participants were excluded from analysis for the following reasons: failure to follow task instructions in the monetary reward task (five subjects), incomplete SCR data (two subjects), technical problems with electrical stimulation (two subjects) and discomfort in the scanner (one subject). Based on literature showing that contingency awareness modulates learning and expression of fear (Grillon, 2002; Jovanovic et al., 2006; Klucken et al., 2009; Tabbert et al., 2011), we further excluded two subjects who had not learned the CS-UCS contingency by the end of the fear acquisition phase. Twenty-six subjects remained in the analysis [mean age (±SD): 23.8 ± 2.4, 16 women]. All participants had normal or corrected-to-normal vision, had not participated in prior fear conditioning experiments involving electric shocks and reported taking no medication affecting the nervous system. Scanner noise protection was provided through foam ear plugs and foam cushions were used to prevent head movement. Participants were reimbursed for their time (€10/h). The study was approved by the local ethics review board and all volunteers gave written informed consent to participate in the study.

### Behavioral task

**Figure 1:**
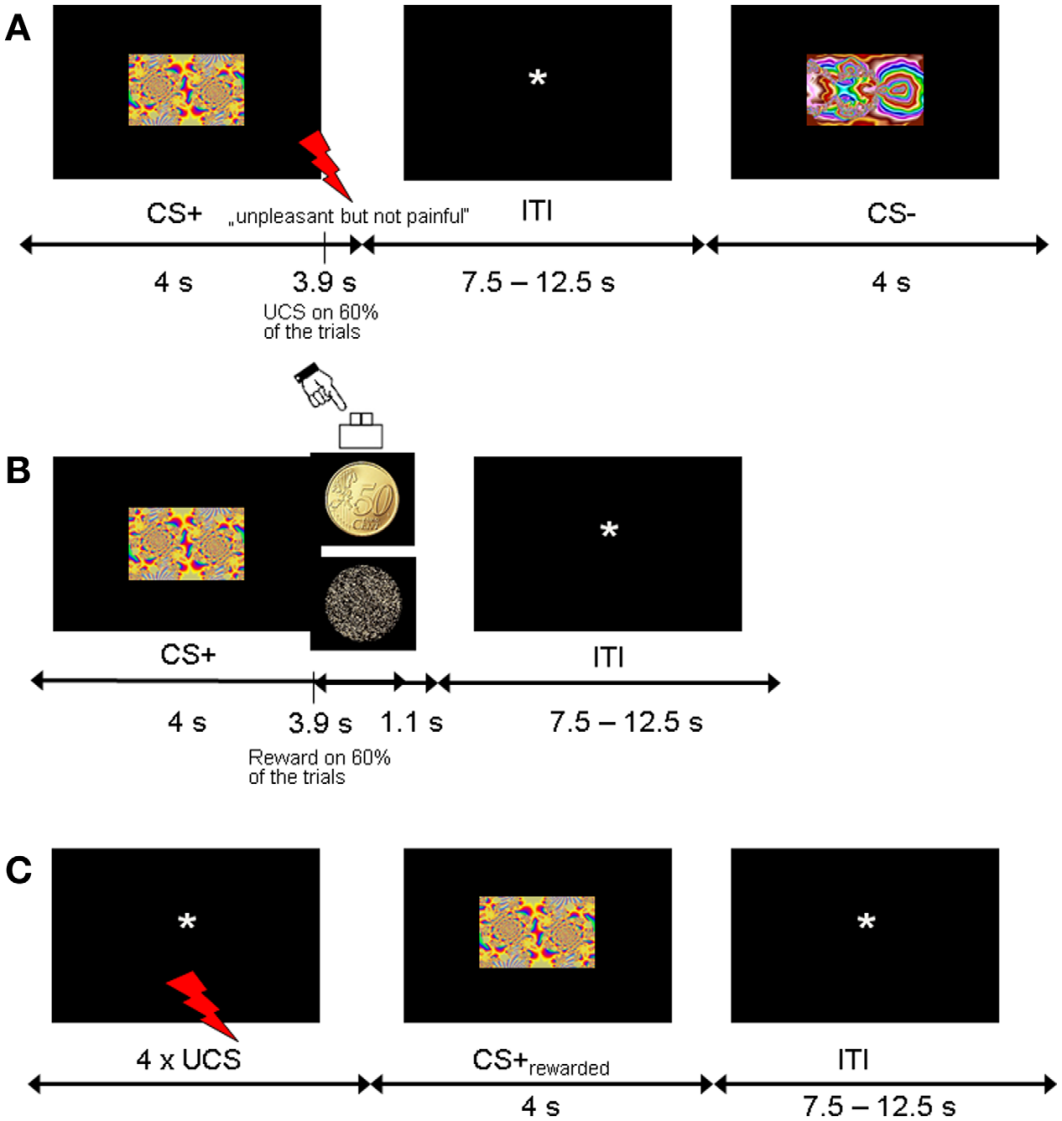
**Experimental design. (A)** Trial sequence for the fear acquisition phase. CS+ were followed by the shock UCS in 60% of trials. **(B)** Trial sequence for the counterconditioning phase. Half of the CS+ and CS− stimuli from the acquisition phase were coupled with monetary reward with 60% contingency, and the other half were never followed by reward. **(C)** Trial sequence for the reinstatement phase. To test for fear recovery, four UCS were unexpectedly given at the beginning of this phase. None of the CSs were followed by shock or reward.

The experiment was divided into three phases: fear acquisition, counterconditioning and reinstatement (Figure 1). Before entering the scanner room, participants were given the instruction for the fear acquisition phase and were shown the money that they could earn later in the counterconditioning phase.

The fear acquisition phase consisted of a Pavlovian conditioning procedure with probabilistic CS-UCS contingencies. The CSs consisted of six different fractal images (4 CS+, 2 CS−, counterbalanced across participants) and the UCS was a mild electric shock to the shin (see below). Participants were told to pay attention to the fractals in order to predict the upcoming electric shocks. There were three consecutive runs of fear acquisition, each of which consisted of 20 CS+ and 10 CS− trials. Each individual fractal image (4 CS+, 2 CS+) was repeated five times per fear acquisition run in random order. CS-UCS contingency was 60%, corresponding to 12 CS+ trials (3 per individual fractal image) coupled with UCS and 8 unreinforced CS+ trials (2 per individual fractal image) per fear acquisition run, yielding a total of 24 unreinforced trials for analysis. Each CS was presented for 4 s at the center of the screen. In reinforced CS+ trials, CS+ presentation coterminated with the UCS. Trials were separated by a fixation phase that was randomly jittered from 7500 to 12,500 ms in 250 ms intervals [mean inter-trial interval (ITI) 10,000 ms]. After completion of the entire fear acquisition phase, participants were asked to rate the unpleasantness of the last electric shock and estimate the CS-UCS contingencies. CSs were presented in random order and participants indicated how often the electrical stimulation followed the shown fractal image (in percent, 0–100% in increments of 10).

In the counterconditioning phase, the six CSs were presented 5 times each in random order in the absence of electric shocks. CS images now served as probabilistic cues predicting rewarded or neutral outcomes. Half of the CS+ and CS− (2 CS+, 1 CS−) indicated reward (with a contingency of 60%) and the other half indicated a neutral outcome, yielding total trial numbers of 10 rewarded CS+, 10 unrewarded CS+, 5 rewarded CS− and 5 unrewarded CS−. Each trial consisted of a CS followed by the outcome (picture of a 50 Eurocent coin or an unrewarded scrambled coin). Participants indicated the outcome type (reward, no reward) by button press (right index or middle finger). In rewarded trials, participants won €0.50 for responding correctly. In unrewarded trials, participants did not win or lose money. No feedback was displayed. Participants were instructed to pay attention to the relationship between different CS and the subsequent outcome type (reward vs. no reward). Each CS was displayed for 4 s at the center of the screen. Outcomes were presented 3.9 s after CS onset and lasted for 1 s after CS offset. Trials were separated by a fixation phase that was randomly jittered from 7500 to 12,500 ms in 250 ms intervals [mean inter-trial interval (ITI) 10,000 ms]. At the end of the counterconditioning phase, participants were asked to estimate the CS-reward contingencies. CSs were presented in random order and participants indicated how often the monetary reward followed the shown fractal image (in percent, 0–100% in increments of 10).

The reinstatement phase immediately followed after counterconditioning. Subjects were briefly told that this experimental session was passive and required no response. Four unexpected presentations of the shock UCS alone were applied at the beginning while a black screen with a fixation cross was shown. There were 24 CS presentations consisting of 8 previously rewarded CS+, 8 previously unrewarded CS+, 4 previously rewarded CS− and 4 previously unrewarded CS− (4 s CS duration; ITI 7500–12,500 ms). No further shocks or rewards were delivered.

### Unconditioned stimuli

Brief transcutaneous electric shocks served as UCS in the fear acquisition phase and as repeated reminder cues at the beginning of the reinstatement phase. A custom-made impulse generator triggered by an optic fiber cable delivered a train of electric pulses (833 Hz, 0.25 ms individual pulse duration) for 100 ms to the middle of the left shin through an Ag/AgCl electrode pair (1 mm^2^ surface each).

The intensity of the electric shock was individually adjusted. In this procedure, each participant was first given a very mild shock which was gradually increased to a level the participant indicated as “unpleasant but not painful.” To assess whether the UCS remained unpleasant throughout the experiment, participants rated the UCS unpleasantness on a 9-point Likert scale (1 = pleasant, 9 = unpleasant) before and after the fear conditioning phase. If the rating was below 7 before the start of the experiment, participants were asked if the UCS intensity could be increased.

### SCR recordings and analysis

SCR were recorded via Ag/AgCl electrodes filled with electrode gel (0.5% NaCl) at a sampling rate of 100 Hz. The electrodes were attached thenar and hypothenar of the left hand. Offline analysis was conducted with the freely available software SCRalyze b2.1.6 (*scralyze.sourceforge.net*) for model-based analysis of peripheral psychophysiology measurements (Bach and Friston, 2013). By formalizing forward models and using an inversion of generative models, this approach infers sympathetic arousal from peripheral psychophysiological signals. We applied the implemented non-linear model, termed dynamic causal modeling (DCM) for event-related SCR, which has been shown to have a higher sensitivity in estimating anticipatory sympathetic arousal compared to conventional (peak scoring) analysis of SCR (Bach et al., 2010a).

SCR were trimmed at the beginning and end of each file (start: 0.9 s before the presentation of the first CS for the fear acquisition and counterconditioning phases and 0.9 s before the presentation of the first UCS for the reinstatement phase; end: 14 s after the last stimulus offset), band pass filtered (1st order butterworth filter with cut off frequencies of 0.0159 and 5 Hz), down sampled to 10 Hz and z-transformed. DCM was based on a canonical response function for SCR (Bach et al., 2010b) and specified on each participant's concatenated experimental runs. To infer sudomotor nerve activity for each individual trial, the onsets and offsets of each event were defined in an unbiased way without differentiating trial types. For the fear acquisition and counterconditioning phases, cue and outcome times were defined in the model. For the reinstatement phase, onsets and offsets of the 4 reminder UCS and the CS were defined. For statistical analysis, we then averaged the inferred peak anticipatory response amplitude for each condition and each participant (Bach et al., 2010a). To avoid potentially confounding effects of the shock on anticipatory SCR estimates, only unreinforced trials (CS+_unreinforced_ and CS−) were included in the statistical analysis. Repeated-measures analyses of variance (ANOVA) were calculated for the counterconditioning and reinstatement phases, with Greenhouse-Geisser corrections where appropriate.

### fMRI recordings and analysis

A 1.5 T whole-body scanner (Siemens Symphony, Erlangen, Germany) with a standard head coil was used to acquire gradient-echo echo-planar images (EPI) at a TR of 2500 ms (TE = 55 ms; slice thickness = 3 mm; gap = 0.6 mm; FoV = 192 mm × 192 mm; flip angle = 90°; matrix size = 64 × 64). 26 slices (voxel size: 3 × 3 × 3 mm) were sampled in descending order at an oblique orientation (−30° to the AC-PC line). Coverage included the whole brain except the most dorsal parts of the parietal lobe. Functional data were acquired in five separate runs (three for fear acquisition, one for counterconditioning and one for reinstatement). A gradient echo field map sequence (TE 1 = 10 ms, TE 2 = 14.76 ms, TR = 1170 ms, 64 slices, voxel size 3 × 3 × 3 mm, matrix size 64 × 64) was recorded before the functional runs to get information for B_0_ distortion correction of the acquired EPI images. Structural images were obtained through a T1-weighted protocol (rapid acquisition gradient echo sequence; 160 sagittal images, 1 mm slice thickness) and a magnetization transfer (MT) sequence with a partial volume that excluded the parietal lobe (80 transversal images, 40 slices, 3 mm slice thickness, 1 × 0.9 × 3 mm voxels).

Imaging data were preprocessed and analyzed with Statistical Parametric Mapping (SPM8, Wellcome Trust Centre for Neuroimaging, University College London, UK) implemented in Matlab. A field map approach was used to calculate and correct for static distortions caused by B_0_ field inhomogeneities. Using the FieldMap toolbox (Hutton et al., 2002), field maps were estimated from the phase difference between the images acquired at the short and long TE. Functional images were then corrected for static geometric distortions and for changes in these distortions due to head motion using the Unwarp toolbox (Andersson et al., 2001). Structural images were segmented for gray and white matter and spatially normalized to the Montreal Neurological Institute (MNI) template. These parameters were then applied to the functional images. Processing of functional images further included realignment, slice time correction (reference slice: 13) and spatial smoothing using an 8 mm Gaussian kernel.

To check the quality of functional images, volume-to-volume variance was calculated using the SPM toolbox TSDiffAna (Brett and Glauche; http://sourceforge.net/projects/tsdiffana.spmtools.p). For statistical analysis, trial-related activity for each participant was modeled by delta functions convolved with a canonical hemodynamic response function. In order to control for low frequency components a high-pass filter with a cut-off value of 128 s was applied and correction for serial autocorrelations was obtained by using first-order autoregressive modeling AR(1). A general linear model (GLM) was specified for each participant to model the effects of interest and six covariates capturing residual motion-related artifacts. For fear acquisition, the three runs were integrated into one model as separate sessions with the following regressors for cues and outcomes: CS+_reinforced_, CS+_unreinforced_, CS−, UCS and no UCS. Reinforced CS+ trials were subsequently excluded from analysis. The model of the counterconditioning phase defined four cue-specific regressors (CS+_rewarded_, CS+_unrewarded_, CS−_rewarded_, CS−_unrewarded_) and two outcome-specific regressors (reward and no reward). For the reinstatement model the following condition-related regressors were entered: UCS, CS+_rewarded_, CS+_unrewarded_, CS−_rewarded_ and CS−_unrewarded_. Statistical parametric maps were generated for each subject from linear contrasts of effects of interest. The relevant contrasts were: CS+ vs. CS− for each phase and CS_rewarded_ vs. CS_unrewarded_ for the counterconditioning phase.

To enable inference at the group level, random-effects analyses were performed using one-sample *t*-tests on contrast images from the first-level analyses. For the counterconditioning and reinstatement phases, we additionally aimed to identify brain areas in which fear association interacted with reward. For this analysis, a repeated-measures ANOVA was calculated on four contrast images per subject in a 2 × 2 flexible factorial design with the factors cue type (CS+, CS−) and reward status (reward, no reward).

Based on our a priori hypotheses, we defined regions of interest and applied small-volume correction (SVC). Except for the amygdala which was anatomically defined using AAL-mask (Tzourio-Mazoyer et al., 2002), regions of interest were defined by centering a sphere on the peak activation coordinates reported by previous studies. Coordinates referring to the (Talairach and Tournoux, 1988) space were first transformed into MNI coordinates using the tal2mni.m script (http://imaging.mrc-cbu.cam.ac.uk/downloads/MNI2tal/tal2mni.m). The radius of each SVC corresponded to the volume of the relevant anatomical structure to correct for an appropriate number of voxels in each structure. The coordinates for activations in the dorsal anterior cingulate cortex (dACC) (*x* = 0, *y* = 12.71, *z* = 36.59) and the insular cortex (*x* = 45.45, *y* = 12.04, *z* = 7.16, and *x* = −30.30, *y* = 22.29, *z* = 8.79) were taken from Phelps et al. (2004) with spheres of 8 mm (Fornito et al., 2008) and 11 mm (Cohen et al., 2010), respectively. For activations in the hippocampus a sphere with a radius of 6 mm (Lupien et al., 2007) was defined around *x* = −22.22, *y* = −15.8, *z* = −16.4 (Phelps et al., 2004). For activations in the vmPFC and OFC, spheres with a radius of 15 mm (Hesslinger et al., 2002; Boes et al., 2009) were defined around *x* = 6.06, *y* = 26.31, *z* = −11.59 (Milad et al., 2007b) and *x* = −4.04, *y* = 29.76, *z* = −19.74 (O'Doherty et al., 2001), respectively. Taking into account that contingency learning has been shown to be associated with the involvement of ventral striatum, we also defined a sphere of 6.6 mm (Anastasi et al., 2006) around the coordinates *x* = 18, *y* = 3, *z* = −3 and *x* = −21, *y* = 9, *z* = −3 (Klucken et al., 2009). For reward-related activations, the coordinates for caudate nucleus (*x* = 9.09, *y* = 8.85, *z* = 9.17) and ventral striatum (*x* = −15.15, *y* = 11.74, *z* = −8.86) were based on Wittmann et al. (2005) with spheres of 9 and 6.6 mm (Anastasi et al., 2006), respectively. Activations in SN/VTA were defined around the coordinates *x* = −12, *y* = −19, *z* = −7 (Guitart-Masip et al., 2011) with a sphere radius of 4.5 mm (Geng et al., 2006).

For better localization of midbrain activity, the relevant activation maps were superimposed on a mean image of spatially normalized MT images acquired from our participants. MT imaging is based on the transfer of energy between protons in free water and highly bound protons within macromolecules (Wolff and Balaban, 1989). Thus MT saturation is thought to be a more direct measure to image myelin and improves contrast between SN and surrounding white matter tracts (Helms et al., 2009) without the geometric distortion present in iron-based imaging such as susceptibility and R2^*^ mapping. It has been shown to allow distinguishing the SN from surrounding structures as a bright area, which has been confirmed to be coextensive with the SN as delineated histologically by tyrosine hydroxylase immunohistochemistry (Bolding et al., 2013). It has also been shown to provide a measure of nigral degeneration in clinical populations such as Parkinson's disease (Eckert et al., 2004; Tambasco et al., 2011).

Significant interaction effects were further evaluated by plotting the mean peak beta values. In order to evaluate the interaction effects between CS type and reward status, we extracted the parameter estimates (beta values) from significant peak voxels and computed a 2 × 2 ANOVA with factors CS type and reward status and appropriate *post-hoc* comparisons. All coordinates are reported in MNI standard space.

## Results

### Behavior

**Figure 2:**
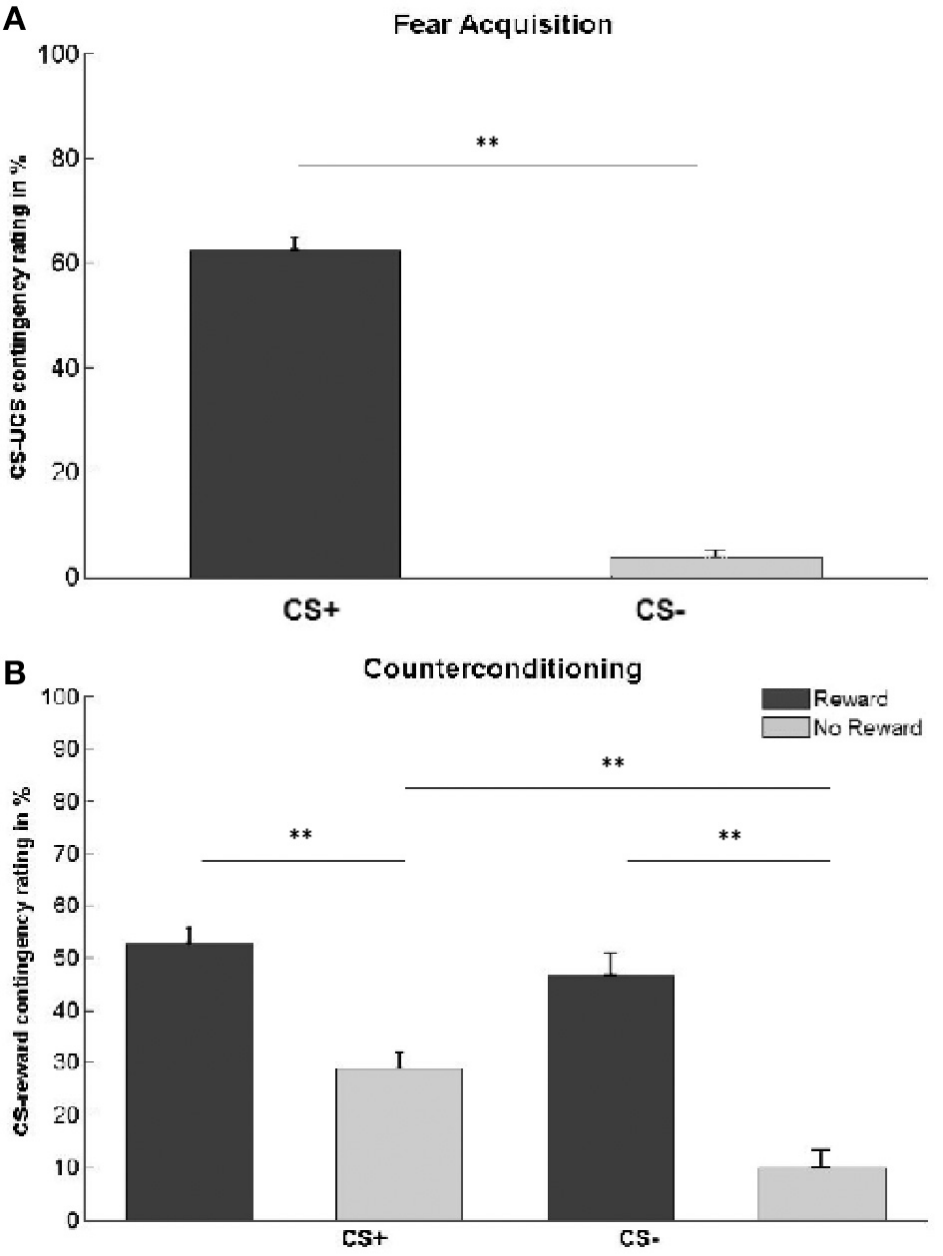
**Contingency awareness ratings. (A)** Subjects' estimation of the percentage of trials in which each CS type was followed by the UCS. Actual contingencies were 60% for CS+, 0% for CS−. **(B)** Subjects' estimation of the percentage of trials in which each CS type was followed by reward. Actual contingencies were 60% for rewarded stimuli (dark gray bars), 0% for unrewarded stimuli (light gray bars). Asterisks indicate significant difference (^**^p < 0.01). Error bars represent standard error.

We first confirmed that the UCS remained aversive throughout the fear acquisition phase. There was no significant difference in mean aversiveness ratings between pre-acquisition (mean ± SE*:* 7.2 ± 0.25) and post-acquisition (6.7 ± 0.33; paired *t*-test, *t*_(23)_ = 1.67, *p* = 0.11). As expected, participants were aware of the CS-UCS contingencies. Estimates of the percentage of trials on which one of the CS+ was followed by the UCS (mean ± SE: 63 ± 2%) were significantly higher than the corresponding mean estimates for the CS− (4 ± 1%; paired *t*-test, *t*_(25)_ = 21.05, *p* < 0.001; Figure 2A).

We also assessed contingency awareness with respect to the CS-reward association in the counterconditioning phase (Table 1, Figure 2B). A 2 × 2 ANOVA with the main factors CS type and reward status yielded significant main effects of CS type [*F*_(1, 25)_ = 12.35, *p* < 0.001], reward status [*F*_(1, 25)_ = 68.21, *p* < 0.001], and a CS type × reward status interaction [*F*_(1, 25)_ = 4.48, *p* = 0.044]. *Post-hoc* comparisons revealed that participants estimated CS+ to have been more often followed by reward than CS− [*t*_(25)_ = 3.51, *p* < 0.001]. This effect was driven by unrewarded stimuli [*t*_(25)_ = 4.94, *p* < 0.001], while no difference was found between rewarded CS+ and CS− stimuli [*t*_(25)_ = 1.15, *p* = 0.26]. Overall, subjects were able to differentiate between rewarded and unrewarded stimuli, independently of whether the CS was previously paired with an electric shock or not [CS_rewarded_ vs. CS_unrewarded:_ *t*_(25)_ = 8.26, *p* < 0.001; CS+_rewarded_ vs. CS+_unrewarded_: *t*_(25)_ = 6.06, *p* < 0.001; CS−_rewarded_ vs. CS−_unrewarded_: *t*_(25)_ = 6.76, *p* < 0.001].

**Table 1:** **Contingency awareness ratings after counterconditioning**.

	**CS-type**		
**Reward status**	**CS+ (%)**	**CS− (%)**	**Overall (averaged) (%)**
Reward	53 ± 3	47 ± 4	50 ± 3
No reward	29 ± 3	10 ± 3	20 ± 3
Overall (averaged)	41 ± 2	28 ± 3	-

*Overview of the means (M) and standard errors (SE) of participants' contingency awareness ratings with respect to CS− reward association in the counterconditioning phase*.

For the counterconditioning phase, a paired *t*-test confirmed that the hit rate in rewarded trials (mean ± SE: 97 ± 1%) was not significantly different from the hit rate in unrewarded trials (97 ± 1%; *t*_(25)_ = 0.18, *p* = 0.86). Because of the partial reinforcement schedule, participants won money on an average of 59 ± 0.6% of rewarded trials. Participants' average reaction time on rewarded hits did not differ from reaction time on unrewarded hits (mean ± SE: reward 610 ± 28 ms; no reward 609 ± 22 ms; *t*_(25)_ = 0.06, *p* = 0.95).

#### SCR

**Figure 3:**
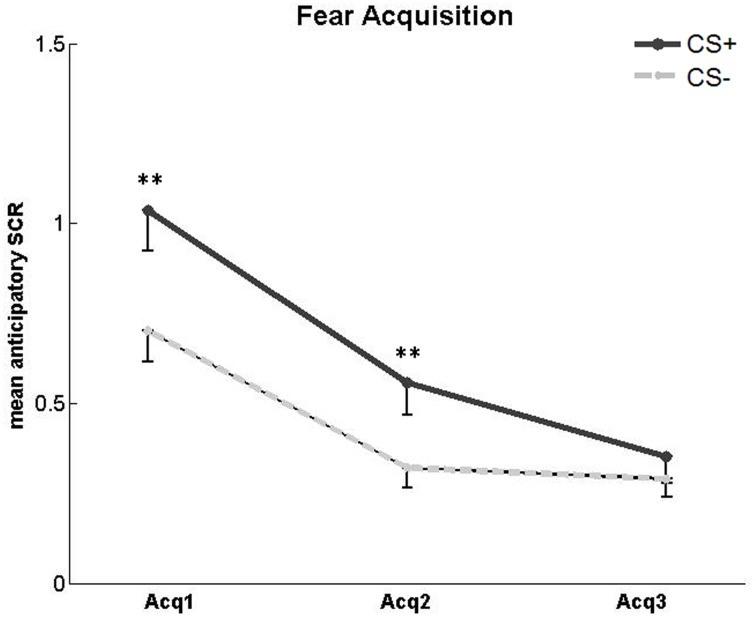
**SCR in the acquisition phase**. Significantly higher mean SCR to unreinforced CS+ compared to CS− in the first and second, but not third, fear acquisition (Acq) run. Asterisks indicate significant difference. Error bars represent standard error.

We first assessed the development of a conditioned SCR during the fear acquisition phase (Figure 3). A 2 × 3 repeated-measures ANOVA with factors CS type (CS+, CS−) and session (first, second and third acquisition run) revealed a main effect of CS type [*F*_(1, 25)_ = 21.5, *p* < 0.001], a main effect of session [*F*_(1.58, 39.53)_ = 26.83, *p* < 0.001], and a CS type × session interaction [*F*_(2, 50)_ = 5.25, *p* = 0.009]. *Post-hoc* paired *t*-tests comparing CS+ vs. CS− responses separately for each run showed a significant difference between CS+ and CS− for the first [*t*_(25)_ = 4.82, *p* < 0.001] and second [*t*_(25)_ = 3.15, *p* = 0.004] fear acquisition run, indicating successful fear learning. In the third fear acquisition run, there was no significant difference in SCR between CS+ and CS− [*t*_(25)_ = 1.14, *p* = 0.27]. This finding is compatible with a habituation of SCR over time as observed in previous studies (Van Ast et al., 2012). To confirm that SCR responses at the end of fear acquisition did not differ with respect to trial types in the subsequent counterconditioning phase, we calculated a 2 × 2 ANOVA with the factors CS type (CS+, CS−) and forthcoming reward status (reward, no reward) for the third fear acquisition run. As expected, there was no main effect of forthcoming reward status and no CS type × reward status interaction.

**Figure 4:**
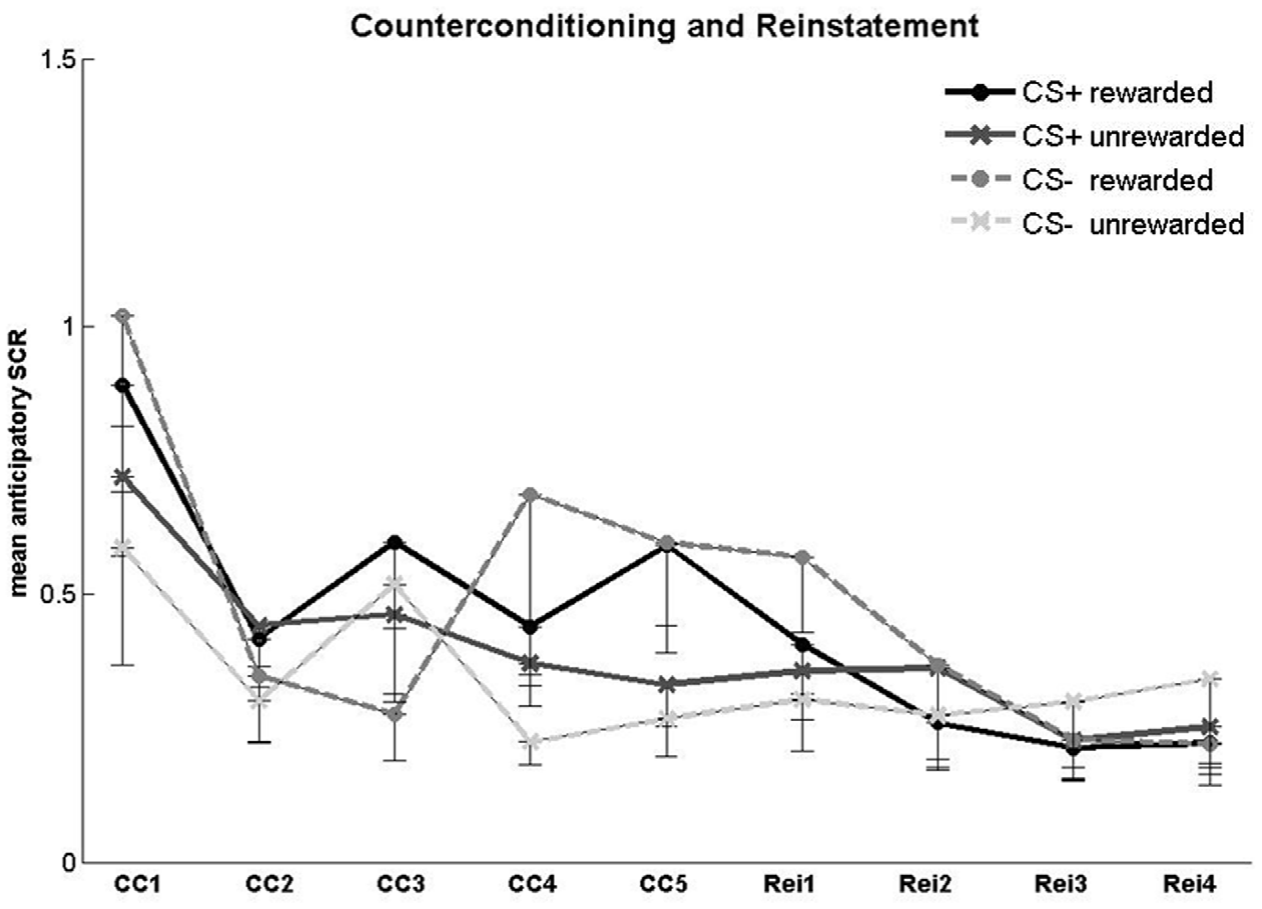
**SCR in the counterconditioning and reinstatement phases**. Anticipatory SCR for each trial in the counterconditioning (CC) and reinstatement (Rei) phase. Error bars represent standard error.

For the counterconditioning phase (Figure 4), we conducted a 2 × 2 × 5 ANOVA with the factors CS type (CS+, CS−), reward status (reward, no reward) and trial (1–5). There was a main effect of reward status [*F*_(1, 25)_ = 8.44, *p* = 0.008], reflecting higher SCR toward rewarded compared to unrewarded CSs (mean ± SE: rewarded 0.59 ± 0.1; unrewarded 0.44 ± 0.06; *t*_(25)_ = 2.7, *p* = 0.01). There was no main effect of CS type and no CS type × reward status interaction. There was a significant main effect of trial [*F*_(3.1, 77.44)_ = 5.64, *p* = 0.001] resulting from an overall SCR decrease across the counterconditioning phase (mean ± SE: first trial 0.8 ± 0.13; last trial 0.45 ± 0.09; *t*_(25)_ = 3.18, *p* = 0.004). No interactions were found between the factors trial and CS type or between the factors trial and reward status.

For the reinstatement phase (Figure 4), a 2 × 2 × 4 ANOVA with the factors CS type, reward status and trial number (1–4) yielded no significant main effects or interactions. Fear recovery following the reminder shocks was assessed in a 2 × 3 ANOVA with the factors CS type (CS+, CS−) and block (second half of the counterconditioning phase, first and second half of the reinstatement phase). Block responses were defined as the mean SCR of the last two counterconditioning trials, the first two reinstatement trials, and the last two reinstatement trials. There were no significant main effects or interactions.

### fMRI results

**Figure 5:**
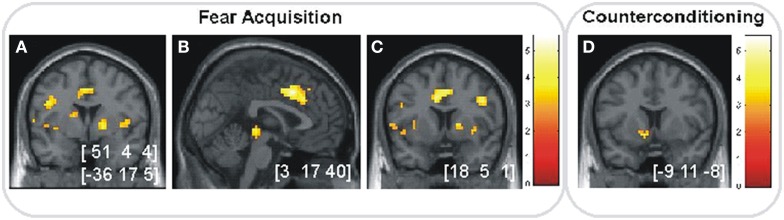
**Anticipation of fear and reward. (A–C)** Significant activations (*p* < 0.05, SVC) in the fear acquisition phase for CS+ vs. CS− in **(A)** bilateral insular cortex, **(B)** right dACC and **(C)** right striatum. **(D)** Significant activation (*p* < 0.05, SVC) in the counterconditioning phase for rewarded vs. unrewarded cues in left ventral striatum. Images are shown in neurological orientation at *p* < 0.005 (uncorrected), for visualization purposes. Color bars indicate *T*-values. Peak coordinates are given in MNI space.

We first confirmed the expected fear and reward effects. In the fear acquisition phase, activation in bilateral insula, right dACC and right ventral striatum was higher for CS+ compared to CS− trials (Figures 5A–C). In the counterconditioning phase, rewarded CSs were associated with higher activation in left ventral striatum compared to unrewarded CSs (Figure 5D).

**Figure 6:**
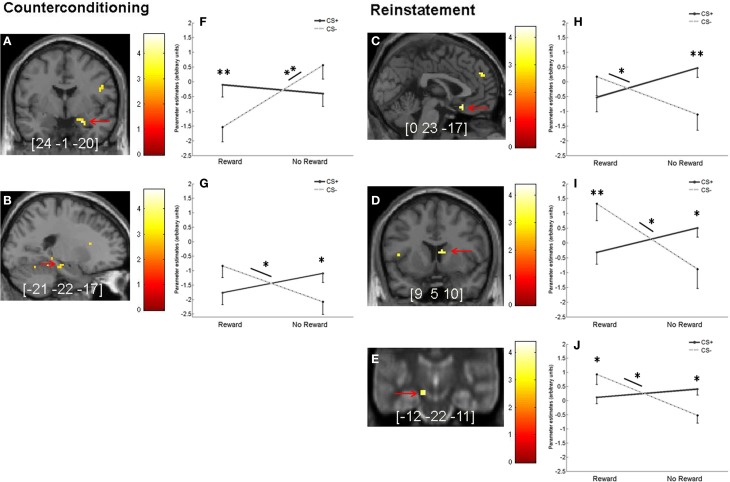
**Interaction of fear and reward during counterconditioning and reinstatement. (A,B)** Interaction between reward status and previous fear association during counterconditioning in **(A)** right amygdala (*p* < 0.05, SVC) and **(B)** left hippocampus (on a trend level, *p* = 0.06, SVC). **(C–E)** Significant interaction (*p* < 0.05, SVC) between reward status and previous fear association during reinstatement in **(C)** OFC, **(D)** right caudate nucleus, and **(E)** left SN/VTA. **(F–J)** Mean parameter estimates (arbitrary units) from the peak voxels of the activations indicated by the red arrows in the left-hand panels broken down by CS type and reward status. To better localize SN/VTA activations, the corresponding panel displays an overlay onto an MT image (cf. methods section). Error bars represent standard error. Images are shown in neurological orientation at *p* < 0.005 (uncorrected), for visualization purposes. Color bars indicate *T*-values. Peak coordinates are given in MNI space. Asterisks indicate significant difference (^*^p < 0.05 and ^**^p < 0.01).

For the counterconditioning and reinstatement phases, we then investigated whether regions in the fear or motivation network showed an interaction of prior fear association and reward learning. For the counterconditioning phase, there were significant interaction effects in amygdala and trend-level effects (*p* = 0.06, SVC) in hippocampus. We then extracted the parameter estimates from both regions and performed *post-hoc* comparisons. For the amygdala (Figures 6A,F), we found a reward-associated signal decrease in CS− trials [*t*_(25)_ = 4.41, *p* < 0.001], while no reward-related difference was found for CS+ trials [*t*_(25)_ = 0.69, *p* = 0.51]. Subsequent pair-wise comparisons showed significantly higher activation for rewarded CS+ compared to rewarded CS− trials [*t*_(25)_ = 2.87, *p* < 0.001] and a trend toward higher activation in unrewarded CS− trials compared to unrewarded CS+ trials [*t*_(25)_ = 1.94, *p* = 0.06]. For the left hippocampus (Figures 6B,G), *post-hoc* comparisons of the extracted parameter estimates revealed that the trend interaction effect was driven by a reward-associated signal increase for CS− [*t*_(25)_ = 2.42, *p* = 0.02] but not for CS+ trials [*t*_(25)_ = 1.64, *p* = 0.11]. Direct comparisons between CS+ and CS− trials showed significantly higher activation for CS+ vs. CS− in unrewarded trials [*t*_(25)_ = 2.33, *p* = 0.02] and a trend toward higher activation for CS− vs. CS+ in rewarded trials [*t*_(25)_ = 1.83, *p* = 0.07].

For the reinstatement phase, there was a significant CS type x prior reward interaction in OFC, right caudate nucleus and left SN/VTA (Figures 6C–E,H,I). For the OFC, *post-hoc* comparisons of the extracted parameter estimates showed a reward-related signal increase for CS− trials [*t*_(25)_ = 2.25, *p* = 0.03] and a trend toward a reward-related signal decrease for CS+ trials [*t*_(25)_ = 2.02, *p* = 0.05]. Further pair-wise comparisons between CS+ and CS− trials revealed a significant difference for previously unrewarded stimuli [*t*_(25)_ = 3.55, *p* < 0.001], but not for previously reward-associated stimuli [*t*_(25)_ = −1.14, *p* = 0.27; Figures 6C,H]. For the caudate nucleus, *post-hoc* tests of the extracted parameter estimates showed a signal increase for previously rewarded compared to unrewarded CS− [*t*_(25)_ = 2.73, *p* = 0.01], but no reward-related difference for CS+ [*t*_(25)_ = −1.72, *p* = 0.10]. We also found significantly higher activation for previously rewarded CS− compared to previously rewarded CS+ [*t*_(25)_ = 3.54, *p* < 0.001], while activation was higher for previously unrewarded CS+ compared to previously unrewarded CS− [*t*_(25)_ = 2.07, *p* = 0.049; Figures 6D,I]. For the SN/VTA, *post-hoc* comparisons of the extracted parameter estimates showed reward-associated activity increase for CS− [*t*_(25)_ = 3.44, *p* < 0.001], but not for CS+ trials [*t*_(25)_ = −1.08, *p* = 0.29]. Direct pair-wise comparisons between CS+ and CS− revealed higher activation for rewarded CS− compared to rewarded CS+ [*t*_(25)_ = 2.24, *p* = 0.03] and higher activation for unrewarded CS+ compared to unrewarded CS− [*t*_(25)_ = 2.61, *p* = 0.02; Figures 6E,J].

## Discussion

Our results show that prior fear conditioning affects subsequent reward learning in a counterconditioning procedure. Specifically, participants were less able to differentiate between rewarded and unrewarded stimuli if they had previously been paired with an aversive shock. This fear-related impairment of reward learning was associated with smaller or absent reward-related amygdala differentiation for CS+ in the counterconditioning phase and with lower activation in the reward network for CS+ compared to CS− in the subsequent reinstatement phase.

Results for the fear conditioning phase confirm that conditioning was successful, as shown by significantly higher shock expectancy ratings, higher SCR, and higher activation in insula and dACC, areas known to be involved in fear conditioning (Büchel et al., 1998; Milad et al., 2007a; Sehlmeyer et al., 2009), for CS+ compared to CS−. In keeping with a recent meta-analysis (Mechias et al., 2010), we did not find significant amygdala activation in the conditioning phase, probably because we employed a mass-univariate fMRI methodology rather than more sensitive multivariate methods (Bach et al., 2011). SCR were found to habituate over time, with significant differences between CS+ and CS− in the first two blocks of the conditioning phase but no significant difference in the third block. This finding is in line with previous reports of SCR habituation to fear cues (Lovibond et al., 2009; Van Ast et al., 2012). The SCR decrease did not result from behavioral habituation to the UCS, as confirmed by the shock aversiveness ratings at the end of the conditioning phase.

The fear association acquired during conditioning then led to a significantly impaired ability to discriminate between rewarded and unrewarded CS+ in the following counterconditioning phase. Specifically, participants overestimated the reward contingency for unrewarded CS+ compared to unrewarded CS−. The higher expectation of reward after CS+ presentation could potentially have resulted from categorization effects. Participants may have generalized contingency information from the fear conditioning phase to the subsequent counterconditioning phase, assigning higher reward-predictive properties to those stimuli that had fear-predictive properties in the acquisition phase. Such generalization processes could have resulted from impaired learning of the new reward-related discriminations for fear-associated stimuli, reconciling the higher reward expectation for unrewarded CS+ with the decreased neural reward effects for CS+ compared to CS− in the counterconditioning and reinstatement phases.

In the course of the counterconditioning phase, there was a significant decrease in SCR for all CS. This decrease could reflect a higher general arousal at the beginning compared to the end of each new experimental phase, in line with reports of arousal-related increases in both tonic and phasic skin conductance components (Barry and Sokolov, 1993). There was no SCR evidence of specific CS+ effects during the counterconditioning phase, consistent with the finding that SCR to the CS+ had already habituated by the last conditioning block. Rewarded stimuli elicited higher SCR compared to unrewarded stimuli, in line with previous studies (Delgado et al., 2008a). In the reinstatement phase, there was no evidence of an effect of prior reward on SCR, and no evidence of a reinstatement of fear responses to the CS+ after reminder shock delivery. A number of studies have reported reinstatement of behavioral and SCR responses in humans when tested in the same session as the acquisition and extinction phases (for a review, see Vervliet et al., 2013). Thus, it is unlikely that the lack of a reinstatement effect in the current study was due to the presentation of all three phases in the course of one session. It is also possible that counterconditioning suppressed subsequent reinstatement not only for rewarded but also for unrewarded CS+. In rats, however, reinstatement effects after counterconditioning have been demonstrated (Brooks et al., 1995). In animal studies, fear conditioning, extinction and reinstatement are usually tested on separate days, allowing for consolidation of the fear and extinction memories (for a review, see Herry et al., 2010). Studies using systematic variations of the delay between acquisition and extinction yielded contrasting results, reporting either stronger (Rescorla, 2004) or weaker (Myers et al., 2006) recovery with short delays. In humans, reinstatement has been demonstrated when acquisition was followed by a consolidation period before extinction and immediate reinstatement testing (Norrholm et al., 2006; Sevenster et al., 2012), when acquisition was immediately followed by extinction and then followed by a consolidation phase (Schiller et al., 2008; Das et al., 2013), with consolidation periods between all three phases (Kindt and Soeter, 2013; Lonsdorf et al., 2013) and when all phases were carried out without consolidation periods (see Vervliet et al., 2013). A future human study systematically comparing the different protocols could be beneficial for theory transfer from animal to human processes. Further difference between animal and human work lies in the use of task instructions. Similar to our protocol, many human studies instruct participants to actively monitor the relationship between cues and shocks (see Sehlmeyer et al., 2009), and a systematic comparison of different human protocols would be helpful.

In the counterconditioning phase, there was an interaction of reward learning and prior fear association in the amygdala. Amygdala activation was lower in reward-associated vs. neutral CS− trials, while there was no difference between reward-related and neutral CS+ trials. Previous studies reported higher activation for CS− compared to CS+ during fear extinction, which was correlated with extinction success (Phelps et al., 2004; Diekhof et al., 2011). This effect was only seen for unrewarded CS− in the current study, suggesting that reward may have interfered with extinction. In turn, prior fear association abolished the differential amygdala activation for rewarded vs. unrewarded stimuli, which was present only in CS− trials. In contrast to the lower amygdala activation for reward-associated vs. neutral CS− in the current study, amygdala activation has previously been found to be higher for stimuli associated with reward (Gottfried et al., 2003; Morrison and Salzman, 2010). Compared to the numerous findings on aversive processing, however, reports of reward-related amygdala activations in fMRI studies have been less frequent. In the current study, the interaction of fear and reward in the same context may have resulted in a changed relative processing of reward in the presence of a safety-signaling CS.

In contrast to the amygdala, the hippocampus showed a strong trend toward an opposite pattern of interaction in the counterconditioning phase, where hippocampal activation was increased by reward in CS− but not CS+ trials. The hippocampus has been shown to be involved in context-dependent fear extinction (for a review, see Ji and Maren, 2007). It is also activated by reward expectation (Schott et al., 2008), represents values and preferences (Lebreton et al., 2009) and is particularly involved when reward learning is based on contextual or spatial properties of the environment (Hölscher et al., 2003; Okatan, 2009). These findings suggest that the interaction pattern in the current study could reflect the diminished reward learning for fear-associated stimuli that was observed behaviorally and in the neural data from the reinstatement phase. Hippocampal activation in the current study was found to be left-lateralized, while amygdala activation was right-lateralized. Previous studies reported more pronounced activity in response to aversive stimuli in the right vs. left amygdala (Vrticka et al., 2013), whereas no lateralization effects have been reported in motivational areas (Diekhof et al., 2012).

The reward network showed an interaction effect in the reinstatement phase, reflecting prior fear and prior reward learning as the CS were not followed by shock or reward in this phase. Activation in OFC, caudate nucleus and SN/VTA was higher for previously rewarded CS− compared to previously unrewarded CS−, while there was no reward-related activity increase in CS+ trials. All three regions are central components of the reward system and known to respond to reward and reward-predictive cues (Haber and Knutson, 2010). The network of striatum and OFC is also involved when reinforcement contingencies change, for example in reversal learning (Frank and Claus, 2006). The observed interaction is therefore consistent with the interpretation that participants had not learned the reward discrimination for stimuli previously paired with shocks by the beginning of the reinstatement phase, which is also supported by the contingency ratings. This is in line with animal experiments demonstrating slower acquisition of appetitive conditioning to fear CS+ (Scavio, 1974; Bromage and Scavio, 1978; Krank, 1985). A previous study investigating the interaction of explicit reward and pain information during choice found an attenuation of reward-related striatal and ACC activity when the outcome of the decision was a mixture of reward and pain (Talmi et al., 2009). Our results suggest that a similar decreased sensitivity to reward information is also found in implicit learning during counterconditioning even when CS+ are no longer followed by shocks.

A possible limitation of this study could lie in the relatively small number of trials. However, because fear learning and extinction processes develop on a short timescale, many studies report relatively small trial numbers, often dividing the phases into early and late components (e.g., Labar et al., 1998; Schiller et al., 2013). In the current study, the significant interaction effects suggest that the task design was sufficient to detect these learning effects. Future studies directly comparing counterconditioning and extinction processes and subsequent reinstatement in humans could provide additional evidence on their similarities and differences.

In conclusion, the present study shows that fear conditioning subsequently impairs the learning of new reward associations to the fear CS+. The amygdala showed an interaction of fear and reward during counterconditioning, while the reward network reflected differences in prior reward learning between fear CS+ and fear CS− in the subsequent reinstatement phase. Our results support the hypothesis that conditioned fear detrimentally affects reward learning during counterconditioning and that this interaction is based on a modulation of areas related to fear processing and reward learning.

### Conflict of interest statement

The authors declare that the research was conducted in the absence of any commercial or financial relationships that could be construed as a potential conflict of interest.
